# Paternal Cardiometabolic Conditions and Perinatal Mortality

**DOI:** 10.1111/ppe.70032

**Published:** 2025-05-20

**Authors:** Shwe Sin Win, Gerhard Sulo, Anders Engeland, Kari Klungsøyr

**Affiliations:** ^1^ Department of Global Public Health and Primary Care Faculty of Medicine, University of Bergen Bergen Norway; ^2^ Department of Chronic Disease Norwegian Institute of Public Health Bergen Norway; ^3^ Department of Health Promotion Norwegian Institute of Public Health Bergen Norway

**Keywords:** cardiometabolic, late miscarriage, paternal health, perinatal mortality, preconception, stillbirth

## Abstract

**Background:**

Studies have suggested that men with cardiometabolic conditions may have an increased risk of offspring perinatal mortality. However, this association remains underexplored.

**Objectives:**

We aimed to study the association between fathers' cardiometabolic conditions and offspring perinatal mortality utilising linked data from national health registries in Norway.

**Methods:**

In this population‐based cohort study, males registered in the Medical Birth Registry of Norway (MBRN), born 1967–2005, were linked to their singleton offsprings born 2004–2020. The Norwegian Patient Registry and the Norwegian Prescription Database were used to define study exposures: history of hypertension, diabetes, dyslipidaemia, severe obesity or any of these at any time before/during the year of childbirth while fathers having no such conditions were the reference group. Perinatal mortality was defined as foetal death from 16 weeks' gestation or neonatal deaths within the first month after birth (from the MBRN). We fitted multilevel random‐intercept Poisson regression models to account for the clustering of infants born to the same father. We reported incidence rate ratio (IRR) with 95% confidence Intervals (CI).

**Results:**

Of 703,746 infants, 3.6% (*n* = 25,314) were born to fathers with any condition. Overall, 4827 (0.7%) of them died perinatally. In fully adjusted models, infants of fathers with hypertension had a 29% higher risk of dying perinatally (IRR 1.29, 95% CI 1.05, 1.57) relative to those of fathers without cardiometabolic conditions. Effect estimates for paternal diabetes, severe obesity and any condition also indicated a possible increased perinatal mortality associated with these conditions. In the sex‐stratified analysis, the associations were stronger in male offspring (IRR 1.29, 95% CI 1.06, 1.58) than female offspring (IRR 1.01, 95% CI 0.78, 1.29).

**Conclusions:**

The increased perinatal mortality in offspring to fathers with cardiometabolic conditions emphasises fathers' biological role in foetal and placental programming and development. Whether offspring sex impacts these associations needs further investigation.

## 1 Background

Studies have indicated that fathers play an essential biological role in foetal and placental programming and development. For example, animal studies have shown that paternal obesity negatively influences blastocyst attachment, growth, and implantation, resulting in reduced placental size and litter size, and disturbed embryo growth and development [1]. Epidemiological studies in humans have also reported associations between insulin resistance, hypertension, and central obesity in fathers and pregnancy complications of placental origin such as impaired placentation, aberrant foetal growth, including intra‐uterine growth restriction, small and large for gestational age (SGA and LGA), gestational hypertension and preeclampsia [2, 3, 4, 5, 6, 7].

Recently, a study reported that the risk of pregnancy loss increased with a higher number of cardiovascular risk factors in fathers [8]. In another study, the same authors reported that fathers with most or all components of the metabolic syndrome (hypertension, hyperglycaemia, dyslipidaemia and obesity) had higher odds of preterm births, low birthweight and longer neonatal intensive care stay compared to fathers without metabolic syndrome [9]. Preterm birth and foetal growth restriction are essential causes of perinatal mortality, and results of these studies indicate a potential association between paternal cardiometabolic conditions and offspring perinatal mortality.

Participants in two of the mentioned studies [8, 9] are based on data from employer‐based, privately insured participants, which makes the studies prone to selection bias, primarily affecting their generalisability to other populations. Moreover, the authors lacked information on the socioeconomic status of the participants, which is an important confounder for the association between fathers' cardiometabolic conditions and perinatal outcomes. Therefore, more research is needed to evaluate these findings in population‐based studies that include participants with varying socio‐economic status.

Given that many lifestyle‐related factors, such as overweight and obesity, are major public health issues with increasing prevalence in adult populations both in Norway and in many other countries, focusing on their impacts is of major interest [10]. We explored the association between fathers' cardiometabolic conditions and offspring perinatal mortality, also taking into account maternal factors and fathers' socioeconomic level.

## 2 Methods

### 2.1 Data Sources

We used linked data from the Medical Birth Registry of Norway (MBRN) [11], the Norwegian Patient Registry (NPR) [12], the Norwegian Prescription Database (NorPD) [13], and the National Education Database (NED) [14], which are all based on mandatory notification to the registries.

The MBRN, established in 1967, is a nationwide registry with information on all live‐ and stillbirths in the country from 16 weeks of gestation [11]. The NorPD contains data about all drugs dispensed to individuals outside institutions since 2004 [13], and the NPR contains person‐identifiable data since 2008 on discharge diagnoses and procedures performed at hospitals and outpatient clinics. NED was established in 1970 and includes information on educational attainment in Norway for all citizens aged 16 years or more [14]. Further details are provided in the Data S1.

### 2.2 Data Linkage

We used the encrypted personal identification numbers (ID) to link data from the MBRN, the NPR, NorPD and NED at the individual level. Males registered in the MBRN and born between 1967 and 2005 were then linked to their singleton infants that could be born during the years 2004 to 2020. The resulting file was a generational file with the birth as the observation unit.

### 2.3 Exposure

Paternal cardiometabolic conditions were the exposures, defined as history of hypertension, diabetes, dyslipidaemia, severe obesity or any of those conditions at any time before/during the year of childbirth. Additionally, we categorised duration of exposures before birth year into ≤ 3, 4–6 or > 6 years. Since most cardiometabolic conditions examined are chronic, a more extended lookback period will indicate that the condition has lasted longer. Fathers with none of these conditions before/during the year of childbirth were the reference group. Information on paternal exposures was available up to 2019 and data on infants up to 2020. For the exposure ‘any cardiometabolic condition’, we stratified the analyses on sex of the offspring (boys and girls).

Patients with uncomplicated hypertension, diabetes and dyslipidaemia are treated in primary care in Norway, and they are not captured in the NPR. In addition, validation studies for some diagnoses in NPR indicate some false positive registrations [15]. The NorPD, on the other hand, captures all prescriptions dispensed from a pharmacy to all individuals in the country, including reimbursement status. Medications given for chronic conditions (e.g., chronic hypertension) are reimbursed in Norway. While medications used for diabetes and dyslipidaemia will mostly be used for their specific indications, antihypertensive medications may also be used for other indications without reimbursement. We therefore used dispensed reimbursed medications from the NorPD to define hypertension, diabetes and dyslipidaemia [16, 17]. This method has also been used previously in other studies from Norway [17]. ATC codes C02, C03, C07, C08, C09 are used to define hypertension, A10 for diabetes and C10 for dyslipidaemia. Obesity is registered only in the NPR (ICD‐10 code E66) and only captures the most severely obese patients where specialist treatment is considered.

### 2.4 Outcome

Outcome was offspring perinatal mortality, defined in our study as intrauterine foetal death from 16 weeks of pregnancy or neonatal death within the first month after birth. We also did separate analyses for stillbirth (defined as intrauterine foetal death from 16 weeks of gestation to birth) and neonatal mortality (first month after birth). We further divided our outcomes into late miscarriage (16–21 gestational weeks) and stillbirth (from 22 weeks). Since early spontaneous abortions (below 16 weeks) are not registered in the MBRN, these were not included in our outcomes. Terminations of pregnancy due to foetal anomaly (which are registered in the MBRN) were excluded.

### 2.5 Covariates

The MBRN provided information on the child's birth year, father's (< 20, 20–24, 25–29 [reference], 30–34, 35–39, 40–44 and ≥ 45 years) and mother's age (< 20, 20–24, 25–29 [reference], 30–34, 35–39, ≥ 40 years) at childbirth, and marital status at childbirth (single or not), mother's chronic hypertension, pregnancy hypertension, pre‐eclampsia, pregestational and gestational diabetes and smoking during pregnancy (all binary).

NED provided information on father's education at 18 or 25 years (depending on father's age at childbirth; defined as primary, high school and college/university education [reference]), and Statistics Norway provided father's annual income at childbirth in Norwegian kroner (low [≤ 250,000], medium low [250,000–402,000], medium [403,000–530,000], high [> 530,001–680,000] and very high [680,001]).

### 2.6 Statistical Analysis

The number of infants each father had varied from one to eleven. These observations are not independent since they cluster in families. Siblings conceived by the same father are exposed to similar genetic, lifestyle and behavioural characteristics of the father that will cause correlations between successive infants of the same father. Moreover, fathers' cardiometabolic conditions could change over their entire reproductive history. The random intercept model accounts for correlation within clusters by separating within group variation from between group variation [18]. Therefore, we fitted multilevel random‐intercept Poisson regression models (father's ID as the random intercept). Incidence Rate Ratios (IRR) and corresponding 95% confidence intervals (CI) were reported, where IRRs correspond to relative risks.

To evaluate whether infants born to fathers with cardiometabolic conditions have increased perinatal mortality independent of maternal risk factors, we repeated analyses in two restricted populations: one where mothers had and in another where mothers did not have specific risk factors of perinatal mortality. We finally evaluated birth weight, gestational age and weight for gestation of the losses to fathers with and without any cardiometabolic condition.

**FIGURE 1:**
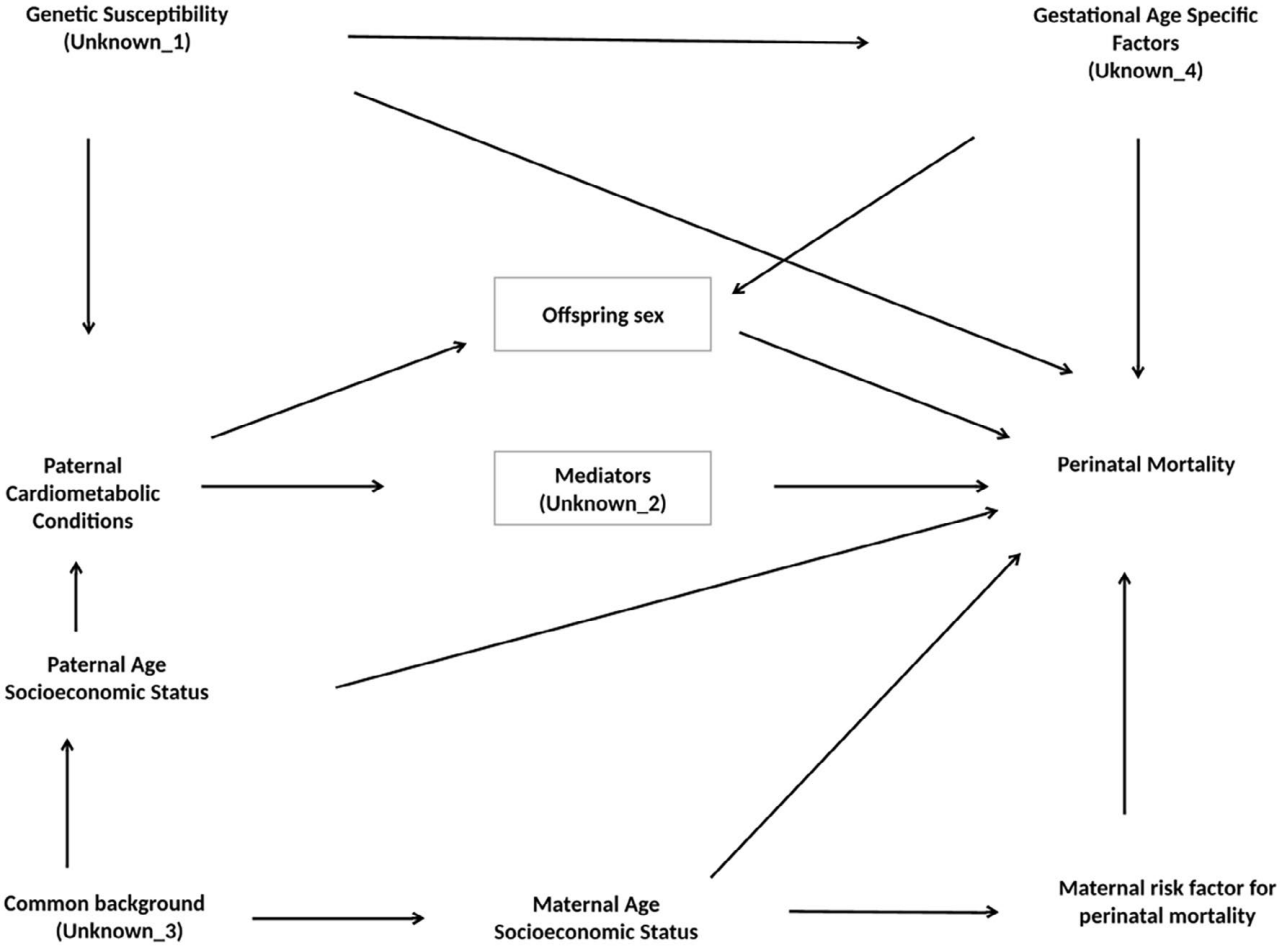
Directed acyclic graph of association between father's cardiometabolic conditions and perinatal mortality. Unknown‐2: For example, epigenetic changes of sperm; Immunological incompatibility; Paternal urogenital tract as cytomegalovirus source. Minimal sufficient adjustment sets for estimating the total effect of paternal cardiometabolic conditions on perinatal mortality: Genetic susceptibility, paternal age and socioeconomic status.

We present results from the unadjusted and fully adjusted models (adjusting for the child's birthyear, father's and mother's age at childbirth, father's income, education and marital status at childbirth, mother's pregestational hypertension and diabetes, mother's preeclampsia, eclampsia, gestational hypertension and diabetes and mother's smoking status during pregnancy). Variables in the models were chosen according to a Directed acyclic graph (DAG) (Figure 1). All analyses were done in STATA version 18.5.

### 2.7 Missing Data

We had no missing values for outcome and exposure variables. Maternal smoking and paternal income had 15.2% and 6.5% missing data, respectively. We did multiple imputations using a logistic regression model for maternal smoking and a multinomial logit regression model for paternal income. Fifty data sets were imputed using all covariates, the outcome variable and the birthweight of the offspring. Other covariates had no or < 5% missing values.

### 2.8 Sensitivity Analyses

In sensitivity analysis, infants were considered exposed only if the year of fathers' diagnosis/prescription of medications was before the year of childbirth to ensure that exposures preceded the outcome.

## Ethics Statement

The study was approved by the Regional Committee for Medical and Health Research Ethics (2020/75421). By law, studies based on registry data alone do not require individual consent.

## 3 Results

### 3.1 Characteristics of the Study Population

**FIGURE 2:**
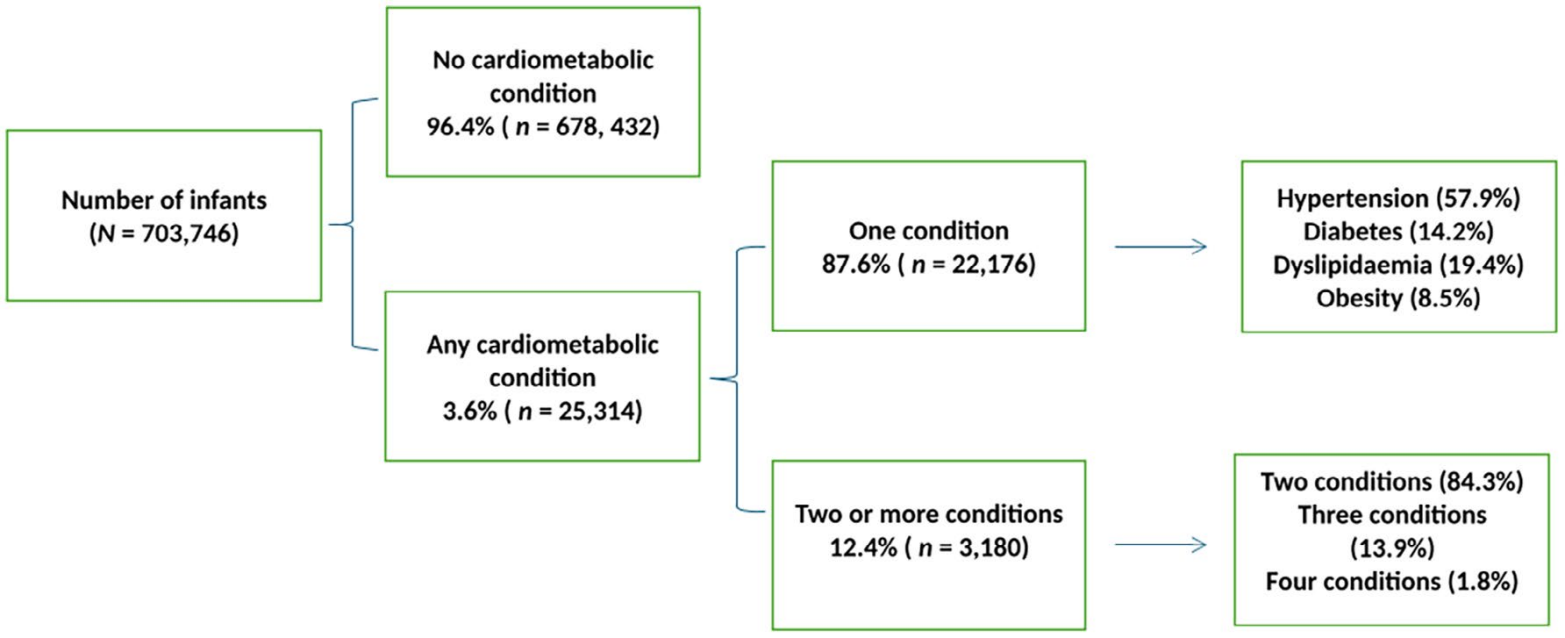
Number of infants born to fathers with and without cardiometabolic conditions (CMD), 2004–2020, Norway.

We analysed 703,746 infants in this study, where 96.4% were born to fathers with none of the specified cardiometabolic conditions, and 3.6% were born to fathers with at least one of these conditions (Table 1). Among infants of affected fathers, 87.6% were born to fathers with only one condition, where hypertension was most common, followed by dyslipidaemia, diabetes, obesity and dyslipidaemia (Figure 2). Overall, 4,827 (0.7%) of infants died during their perinatal period (Table 1).

**TABLE 1:** Characteristics of fathers to offspring born 2004–2020, Norway: Overall and by presence of cardiometabolic conditions.

	Total	No cardiometabolic conditions	Any cardiometabolic conditions^a^
Number of births, *n* (%)	703,746	678,432 (96.4)	21,314 (3.6)
Number of perinatal deaths	4,827 (0.7)	4,617 (0.7)	210 (0.8)
Age at childbirth (year), *n* (%)			
< 20	3,994 (0.6)	3,925 (0.6)	69 (0.3)
20–24	49,990 (7.1)	48,026 (7.2)	964 (3.8)
25–29	174,353 (24.8)	170,394 (25.1)	3959 (15.6)
30–34	256,121 (36.4)	248,807 (36.7)	7314 (29.0)
35–39	163,475 (23.2)	156,194 (23.0.2)	7281 (28.8)
40–44	55,810 (7.9)	50,083 (7.4)	5727 (22.6)
Education			
Basic Education	128,900 (18.3)	122,554 (18.1)	6346 (25.1)
High school	353,177 (50.2)	340,398 (50.2)	12,799 (50.5)
University	213,909 (30.4)	208,046 (30.7)	5863 (23.2)
Missing	7760 (1.1)	7434 (1.1)	326 (1.3)
Income in NOK, *n* (%)			
Very Low (≤ 250,000)	96,439 (13.7)	91,797 (13.5)	4642 (18.3)
Low (> 250,000 to < 402,000)	215,411 (30.6)	207,092 (30.5)	8319 (32.9)
Medium (> 402,000 to ≤ 530,000)	170,047 (24.2)	164,535 (24.3)	5512 (21.8)
High (> 530,000 to ≤ 680,000)	105,692 (15.0)	102,455 (15.1)	3237 (12.8)
Very high (> 680,000)	70,967 (10.1)	69,054 (10.2)	1913 (7.6)
Missing	45,190 (6.4)	43,499 (6.4)	1691 (6.7)

^a^ Includes infants of fathers with hypertension, diabetes or dyslipidaemia registered from 2004 to 2019 and obesity from 2008 to 2019.

A higher proportion of affected fathers were in the older age groups at childbirth compared to unaffected fathers (Table 1). A lower proportion of affected fathers had college/university education and higher or very high incomes than their counterparts.

### 3.2 Associations Between Father's Cardiometabolic Conditions and Perinatal Death

**FIGURE 3:**
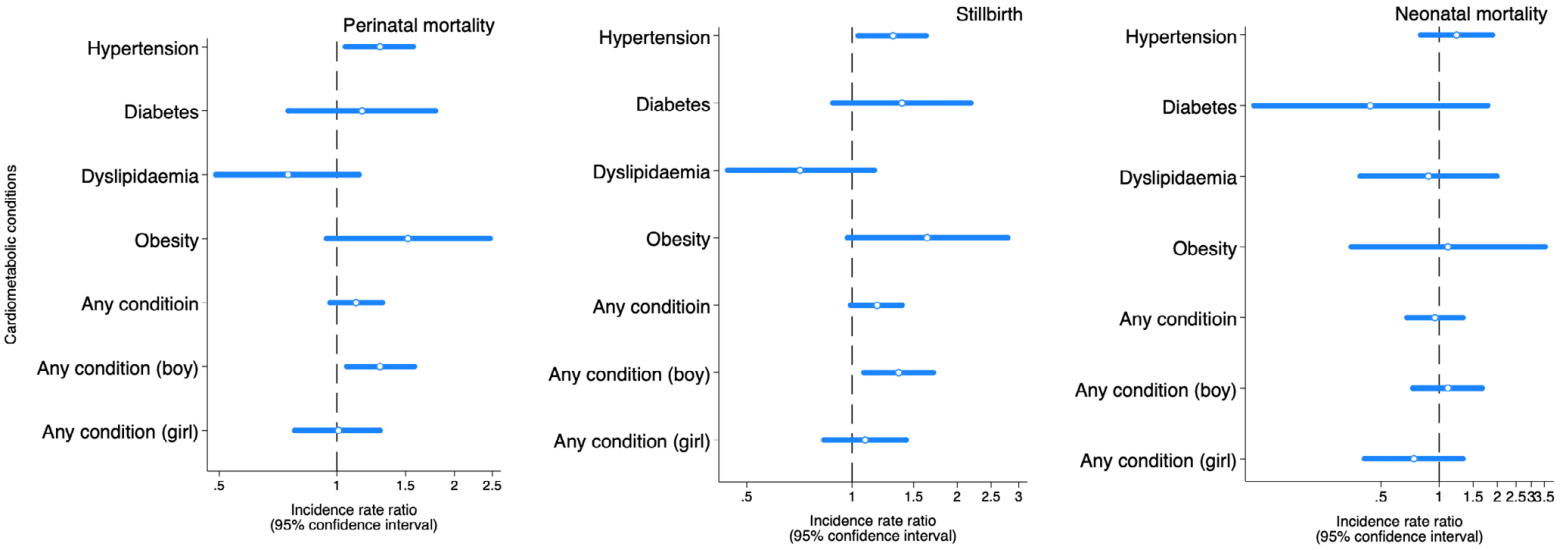
Associations between father's cardiometabolic conditions and perinatal mortality in their offsprings born 2004–2020, Norway. Perinatal mortality: Intrauterine foetal death from 16 weeks of pregnancy or neonatal death within the first month after birth. Stillbirth: Intrauterine foetal death from 16 weeks of gestation to birth. Neonatal mortality: Death within the first month after birth.

In the fully adjusted model, infants of fathers with hypertension had a 29% higher risk of dying perinatally (IRR 1.29, 95% CI 1.05, 1.57) than those of unaffected fathers (Figure 3 and Table S1, model 2). Point estimates for paternal diabetes, severe obesity and any condition also indicated a possible increased perinatal mortality associated with these conditions.

Post‐estimation intra‐class correlations for each exposure were 47% for hypertension and diabetes, 53% for dyslipidaemia, 55% for obesity and 53% for any condition. Therefore, around 50% of the variations in perinatal mortality can be explained by the individual father's genetic, lifestyle and behavioural characteristics.

In offspring sex‐stratified analysis, fathers with any condition were associated with perinatal mortality, which was only present for boys (Figure 3 and Table S1). In the case of duration of exposure, infants of fathers who dispensed their first antihypertensive medication ≤ 3 years before childbirth had the highest perinatal mortality risk, while for diabetes, obesity or any cardiometabolic condition, the highest point estimate was found when the first registered medication or diagnosis was 4–6 years before childbirth (Table 2). However, for all exposures, confidence intervals were wide and overlapping across the lookback periods.

**TABLE 2:** Association between father's cardiometabolic conditions and perinatal mortality categorised by duration of exposure. Norway, 2004–2020.

Exposure	Total (number)	Perinatal deaths number (%)	Incidence rate ratio (95% confidence interval)^a^	
			Unadjusted	Adjusted
Hypertension				
No	688,289	4692 (0.7)	1.00 (Reference)	1.00 (Reference)
≤ 3 year	9043	91 (1.0)	1.43 (1.16, 1.78)	1.37 (1.10, 1.70)
4–6 year	3653	28 (0.8)	1.10 (0.73, 1.78)	1.09 (0.72, 1.64)
> 6 year	2761	16 (0.6)	0.85 (0.53, 1.38)	0.89 (0.55, 1.44)
Diabetes				
No	699,169	4795 (0.7)	1.00 (Reference)	1.00 (Reference)
≤ 3 year	2323	11 (0.5)	0.69 (0.38, 1.24)	0.67 (0.38, 1.21)
4–6 year	1253	13 (1.0)	1.55 (0.88, 2.73)	1.66 (0.94, 2.94)
> 6 year	1001	8 (0.8)	1.17 (0.54, 2.51)	1.32 (0.61, 2.87)
Dyslipidaemia				
No	697,568	4786 (0.7)	1.00 (Reference)	1.00 (Reference)
≤ 3 year	4098	25 (0.6)	0.86 (0.58, 1.28)	0.78 (0.52, 1.16)
4–6 year	1120	9 (0.8)	1.00 (0.52, 1.92)	0.92 (0.48, 1.77)
> 6 year	1289	9 (0.7)	1.24 (0.56, 2.75)	1.15 (0.52, 2.56)
Obesity^b^				
No	541,989	3529 (0.7)	1.00 (Reference)	1.00 (Reference)
≤ 3 year	1648	13 (0.8)	1.18 (0.67, 2.09)	1.14 (0.64, 2.02)
4–6 year	635	9 (1.42)	2.14 (1.11, 4.11)	1.61 (0.77, 3.40)
> 6 year	506	5 (1.10)	1.45 (0.54, 3.86)	1.59 (0.60, 4.22)
Any CMD^c^				
No	678,432	4617 (0.7)	1.00 (Reference)	1.00 (Reference)
≤ 3 year	13,278	112 (0.8)	1.21 (1.00, 1.46)	1.16 (0.96, 1.41)
4–6 year	7498	66 (0.9)	1.28 (0.99, 1.65)	1.22 (0.94, 1.59)
> 6 year	4538	32 (0.7)	1.03 (0.71, 1.48)	1.09 (0.75, 1.59)
Any CMD (boys)^c^				
≤ 3 year	6716	62 (0.9)	1.34 (1.03, 1.73)	1.28 (0.98, 1.67)
4–6 year	3824	39 (1.0)	1.49 (1.07, 2.08)	1.42 (1.01, 2.01)
> 6 year	2303	18 (0.8)	1.14 (0.71, 1.83)	1.26 (0.78, 2.03)
Any CMD (girls)^c^				
≤ 3 year	6549	37 (0.6)	1.02 (0.74, 1.42)	0.98 (0.70, 1.35)
4–6 year	3669	22 (0.6)	1.09 (0.71, 1.69)	1.04 (0.67, 1.63)
> 6 year	2235	14 (0.6)	1.13 (0.63, 2.02)	1.15 (0.64, 2.07)

Abbreviation: CMD, cardiometabolic conditions as defined by medication or diagnosis before/during the year of childbirth.

^a^ Multilevel random‐intercept Poisson regression (father's identification number as the random‐intercept). Adjusted IRR: adjusted for birthyear of child; father's and mother's age at childbirth; father's income, education and marital status at childbirth; mother's pregestational hypertension and diabetes; mother's preeclampsia, eclampsia, gestational hypertension and diabetes; and mother's smoking status during pregnancy.

^b^ Includes infants of fathers with obesity registered from 2008 to 2019.

^c^ Includes infants of fathers with hypertension, diabetes or dyslipidaemia registered from 2004 to 2019 and obesity from 2008 to 2019.

In the restricted population where mothers with important risk factors for perinatal mortality were excluded, the point estimates for the association between father's cardiometabolic conditions and offspring perinatal mortality became stronger than in the population where only mothers with risk factors were included (Table S2). However, all the 95% confidence intervals overlapped across the two study populations for all the exposures, highlighting that paternal associations were independent of maternal risk factors.

When splitting our outcome into stillbirth and neonatal mortality, point estimates were slightly higher for stillbirth but less precise than perinatal mortality (Figure 3, Table S3 and Table S4). In case of late miscarriage (from 16 to 21‐week gestation), the estimates were even higher than stillbirth but less precise (Table S5).

The associations were similar in the sensitivity analysis where infants were considered exposed only if the year of fathers' diagnosis/dispensed medications was before their infants' birth year (Table S7).

### 3.3 Characteristics of Infants Who Had Died in the Perinatal Period

A total of 4827 infants died perinatally (Table S8). The mean birthweight was slightly higher in losses where fathers with versus without cardiometabolic conditions. SGA at the 10th percentiles was more common in losses to fathers with than without conditions. As for gestational age, the two extremes, that is, extremely preterm (≤ 31 weeks) and post‐term (≥ 42 weeks), were more common in losses to fathers with than without cardiometabolic conditions.

## 4 Comment

### 4.1 Principal Findings

Using linked data from population‐based registries in Norway, we have shown that infants of fathers with cardiometabolic conditions are at higher risk of perinatal mortality than those born to fathers without such conditions independent of maternal risk factors. Similar patterns were seen for risk of late miscarriage and stillbirth. Among the specific conditions, paternal hypertension was associated with higher risk of perinatal mortality. When stratifying by offspring sex, the association was only evident for boys.

### 4.2 Strengths of the Study

This study used linked data from population‐based nationwide registries with mandatory notification and included 703,746 observations enabling us to study a rare outcome without selection bias and where we could adjust for father's socioeconomic level, a potential confounder. The MBRN has complete coverage of livebirths due to routine automatic linkage with the National Population Register as well as specific quality tasks in place to ensure good ascertainment for pregnancy losses from 16 weeks onward. The definition of hypertension, diabetes and dyslipidaemia was based on dispensed and reimbursed medication, which provides good validity for those needing medication.

### 4.3 Limitations of the Data

We lacked data for some maternal factors for adjustment, for example, maternal BMI and education, both associated with perinatal mortality. However, we did adjust for the mother's age, pregestational and gestational hypertension, pregestational and gestational diabetes, smoking habits and the fathers' education and income, which are all correlated to the mothers' BMI and education. There may, however, still be residual confounding.

We captured pregnancy losses and stillbirths from 16 completed weeks of gestation. Our results can therefore not be generalised to fathers who experience earlier pregnancy losses. Hence, prospective cohort studies that can capture implantation failure and early pregnancy loss would also be interesting. When stratifying by offspring sex, collider stratification could occur, as shown in our DAG (Figure 1). An unknown factor that affects the gestational age specific losses by sex could be a source of such collider bias, since our data do not cover early losses. We therefore looked at the distribution of gestational age in male and female losses and found that male losses were more likely to be very preterm (≤ 31 weeks of gestation) than female losses regardless of paternal cardiometabolic conditions. Therefore, we suggest that other unknown biological mechanisms may lead to a higher vulnerability in males than females independent of gestational age, with higher mortality if exposed to factors such as (in our study) fathers' cardiometabolic conditions. However, we cannot exclude that collider stratification bias could slightly affect our estimates when we stratify on offspring sex.

Moreover, since the timing of fathers' cardiometabolic conditions is defined as first dispensed medication before/during the year of childbirth, some medications could have been dispensed after conception. However, the associations were similar when we restricted the timing of exposures to the years before the birth year.

In the MBRN, stillbirths have more missing fathers' ID than livebirths. When studying fathers and risk of perinatal mortality, this can be a problem in our study population since we use the father's ID when linking fathers to their offspring. While mother's IDs are captured through routine automatic linkage with the Population Registry for all births, father's IDs for stillbirths are manually collected and there is therefore a higher risk of missing data. During our study years, 11% of stillbirths had missing IDs for fathers and these stillbirths were thus not captured in the father‐child linkage. For late miscarriage, the proportion of missing fathers' IDs was even higher. However, the missing is random and not associated with any other information about the fathers, and it is therefore not a likely source of selection bias. It will, on the other hand, dilute our stillbirth rates since there is a lower number of losses in the second generation when linking fathers to their offspring compared to what is found for rates in general.

Obesity is registered only in NPR that captures the most severely obese patients, our results may therefore not be generalizable to all obese fathers. When analysing ‘any condition’, fathers with severe obesity will not be captured for births in 2004–2007, and these fathers will therefore be misclassified as unaffected during these years. However, the proportion of obesity in the ‘any cardiometabolic condition’ for these years was only 15%. We therefore suggest that the misclassification of paternal obesity for births in 2004–2007 in ‘any condition’ is too small to impact our results.

We were unable to investigate a potential dose–response relationship due to the limited number of fathers with two or more conditions and very few losses among these. The possible explanation may be that our fathers are relatively young compared to men with cardiometabolic conditions in general, and also that those with two or more conditions may be less fertile [19]. Moreover, we could not categorise our exposures graded by severity and therefore could not evaluate effect modification by the severity of the exposure. More extensive studies are needed to categorise the severity of exposures and the number of conditions, allowing for the investigation of a potential dose–response relationship.

## 5 Interpretation

Our study's findings align with those of published international studies. Kasman and colleagues [8] reported that fathers with any components of the metabolic syndrome had a 10% higher risk (RR 1.10, 95% CI 1.09, 1.12) of experiencing pregnancy loss compared to fathers with no components of the metabolic syndrome. In another study, the same authors reported that fathers with metabolic syndrome had higher odds of having preterm birth, infant low birth weight and longer neonatal intensive unit stay compared to fathers without such conditions regardless of maternal factors and maternal smoking [9].

Several possible mechanisms can explain a paternal role in conception, embryonic development and pregnancy loss. First, paternally derived foetal genes following both Mendelian law and genomic imprinting can influence the implantation process, vascular modelling of the fetoplacental unit, thrombosis and clotting of the placental vasculature [20], placentation and subsequent placental function [21, 22]. Similarly, maternal exposure to immune‐incompatible antigens from the paternal semen may trigger a cascade of immunological reactions leading to placental rejection, impaired implantation and influence placental morphogenesis with subsequent dysfunction of the placenta [21, 22, 23].

Epigenetic changes of sperm include sperm DNA fragmentation, which makes it more susceptible to environmental and metabolic insults [24]. This can result in abnormal gametes with unbalanced genetic material due to chromosome abnormalities, both in terms of number and structure, Y chromosome microdeletion, aneuploidy in the embryo's genes, and subsequent abnormal early development [21]. Sperm DNA fragmentation could also lead to deviations in sperm proteomics play a pivotal role in fertilisation and the early stages of embryonic development [24, 25].

Finally, the male urogenital tract is the major reservoir for cytomegalovirus, which can easily change the cytokine levels in seminal fluid, which again may trigger a cascade of cellular, molecular and immunological reactions influencing maternal tolerance or intolerance toward the foetus [26].

We found that the risk of perinatal mortality associated with paternal cardiometabolic conditions was higher for male offspring and not evident for female offspring, although confidence intervals overlapped. This finding should be investigated further. Studies have shown that the presence of a male foetus generates more pro‐inflammatory cytokines and less anti‐inflammatory cytokines in the trophoblast than a female foetus [27]. It might be explained by sex playing a role in foetal programming. Animal models of foetal programming proved that male offspring are more sensitive to insults during development than female offspring [28]. This has been reported from animal studies and human studies and may be related to sex hormones [28]. There is therefore a potential biological basis for paternal cardiometabolic conditions affecting the risk of perinatal mortality differently in male and female offspring; however, these findings warrant further study.

Among infants who died perinatally, those whose fathers had cardiometabolic conditions had slightly higher mean birth weight. Still, they were more likely to be small (SGA at 10th percentile) and to be in the two extreme gestational ages than those whose fathers were without cardiometabolic conditions. Our finding of SGA being more common in fathers with cardiometabolic conditions is in line with the literature [2, 4, 6].

Improving fathers' preconception health may positively influence pregnancy outcomes, with impacts extending to broader biological contributions to the health of their offspring [29]. A systematic review has shown that primary care‐based preconception interventions improve health knowledge and reduce preconception risk factors in mothers. The same study highlighted the limited evidence for fathers in the same area, indicating a need for further research [30]. Thus, the findings of the present study contribute to the growing evidence of the importance of paternal health for pregnancy outcomes and highlight the value of optimising paternal health before conception.

## 6 Conclusions

Evidence for the father's role in pregnancy and perinatal health is recently growing but has yet to be recognised by the broader clinical and research communities. We have shown that fathers with cardiometabolic conditions are at higher risk of offspring perinatal mortality than fathers without such conditions, independent of maternal risk factors. These associations were stronger for male offspring, highlighting the potential influence of paternal cardiometabolic conditions on perinatal mortality in a gender‐specific manner and are a finding that should be investigated further.

## Author Contributions

K.K. and S.S.W. conceived and designed the study. K.K. received funding and obtained access to data. A.E. linked the data. S.S.W. conducted formal analysis and drafted the initial version of the manuscript. K.K. and G.S. provided supervision. All the authors revised the manuscript.

## Ethics Statement

The study was approved by the Regional Committee for Medical and Health Research Ethics (2020/75421).

## Consent

Studies based on registry data alone do not require individual consent, by law.

## Conflicts of Interest

The authors declare no conflicts of interest.

## Supporting information


Data S1.

Tables S1–S8.

## Data Availability

The data used in this study is defined as potentially identifiable based on type, number of variables and family/generational linkage. They also include sensitive information such as perinatal mortality and parental health issues. In Norway, ethical and legal restrictions exist for sharing potentially identifiable datasets: ACT 2008‐06‐20 no. 44: the Health Research Act and GDPR‐ General Data Protection Regulation. However, data are available for other researchers upon request to the Norwegian Institute of Public Health (https://helsedata.no/) after approval from the Regional Ethic Committee (ethical consent: https://rekportalen.no/#hjem/home).
