# Supplementary material for: Paternal Cardiometabolic Conditions and Perinatal Mortality
Source: Paediatr Perinat Epidemiol. 2025 May 20;39(6):559–67. doi: 10.1111/ppe.70032 (PMC12391856; doi:10.1111/ppe.70032)
Supplement: Supplementary file 1 — Data S1. [file PPE-39-559-s001.docx]

Description of the registries and accessibility of health care services and pharmaceuticals in Norway

The Norwegian health care system is universally financed by taxation, income-related contributions and co-payments. ^1^

Medical Birth Registry of Norway

The MBRN is a nationwide registry based on compulsory notification of all live- and stillbirths in the country from 16 weeks of gestation (12 weeks since 2002). ^2^ It was established in 1967 and collects information through standardized forms including maternal health before and during pregnancy, complications during pregnancy and delivery, interventions during labor, and mother’s and child’s health after birth including neonatal diagnoses. Information on maternal smoking during pregnancy has been collected since 1999, but mothers can opt out from registering this factor.

Norwegian Prescription Database

Diagnoses and procedures from specialist care will be captured in the Norwegian Patient Registry, while diagnoses and procedures from primary care will not. The Norwegian Prescription Database captures all prescribed medications dispensed to individuals from any pharmacy in the country, including information on reimbursement. The medications are registered using the Anatomical Therapeutic Chemical (ATC) classification system.^3^ Medications that are only taken during a hospital stay will not be captured.^4^”.

Norwegian Patient Registry

The NPR is a registry containing information on discharge diagnoses and procedures performed at hospitals and out-patient clinics.^5^ Diagnoses are registered by ICD codes (at present version 10). NPR was established in 1997 but has only been person-identifiable since 2008.

National Education Database

NED ﻿was established in 1970 and contains information on educational attainment in Norway for all citizens aged16 years or more.^6^

References

1. Laugesen K, Ludvigsson JF, Schmidt M, Gissler M, Valdimarsdottir UA, Lunde A, et al. Nordic Health Registry-Based Research: A Review of Health Care Systems and Key Registries. CLEP. 2021 Jul 19;13:533–54.

2. Irgens LM. The Medical Birth Registry of Norway. Epidemiological research and surveillance throughout 30 years. Acta Obstetricia et Gynecologica Scandinavica. 2000;79(6):435–9.

3. ATC Code [Internet]. [cited 2025 Jan 7]. Available from: https://www.atccode.com/

4. Furu K. Establishment of the nationwide Norwegian Prescription Database (NorPD) – new opportunities for research in pharmacoepidemiology in Norway.

5. Norwegian Institute of Public Health. Norwegian Patient Registry (NPR) [Internet]. [cited 2024 Jun 24]. Available from: https://helsedata.no/en/forvaltere/norwegian-institute-of-public-health/norwegian-patient-registry-npr/

6. Statistics Norway. Educational attainment of the population [Internet]. SSB. [cited 2024 Jun 24]. Available from: https://www.ssb.no/en/utdanning/utdanningsniva/statistikk/befolkningens-utdanningsniva
