# Supplementary material for: Paternal Cardiometabolic Conditions and Perinatal Mortality
Source: Paediatr Perinat Epidemiol. 2025 May 20;39(6):559–67. doi: 10.1111/ppe.70032 (PMC12391856; doi:10.1111/ppe.70032)
Supplement: Supplementary file 2 — Tables S1–S8. [file PPE-39-559-s002.docx]

eTable1. Association between father’s cardiometabolic conditions and offspring perinatal mortality. Norway, 2004-2020

| Exposure | | Total (N) | Perinatal Deaths, n (%) | IRR ^a^ | |
| --- | --- | --- | --- | --- | --- |
| Hypertension | |  |  | IRR* (95%CI) | IRR** (95%CI) |
|  | No | 678,432 | 4,617 (0.7) | 1.00 (Reference) | 1.00 (Reference) |
|  | Yes | 12,833 | 118 (0.9) | 1.33 (1.10, 1.60) | 1.29 (1.05, 1.57) |
| Diabetes | |  |  |  | |
|  | No | 678,432 | 4,617 (0.7) | 1.00 (Reference) | 1.00 (Reference) |
|  | Yes | 3,151 | 23 (0.7) | 1.09 (0.71, 1.65) | 1.16 (0.75, 1.79) |
| Dyslipidaemia | |  |  |  | |
|  | No | 678,432 | 4,617 (0.7) | 1.00 (Reference) | 1.00 (Reference) |
|  | Yes | 4,311 | 28 (0.7) | 0.93 (0.64, 1.33) | 0.75 (0.49, 1.14) |
| Obesity ^e^ | |  |  |  | |
|  | No | 518,256 | 3,346 (0.7) | 1.00 (Reference) | 1.00 (Reference) |
|  | Yes | 1,881 | 21 (1.1) | 1.69 (1.06, 2.68) | 1.52 (0.94, 2.47) |
| Any CMD ^m^ | |  |  |  | |
|  | No | 678,432 | 4,617 (0.7) | 1.00 (Reference) | 1.00 (Reference) |
|  | Yes | 25,314 | 210 (0.8) | 1.20 (1.04, 1.38) | 1.12 (0.96, 1.31) |
|  | Boys | 12,843 | 119 (0.9) | 1.35 (1.11, 1.63) | 1.29 (1.06, 1.58) |
|  | Girls | 12,453 | 73 (0.6) | 1.06 (0.83, 1.36) | 1.01 (0.78, 1.29) |

CMD: cardiometabolic conditions as defined by medication or diagnosis before/during the year of childbirth

^a^ multilevel random-intercept Poisson regression (father's identification number as the random-intercept)

IRR* crude

IRR** adjusted for birthyear of child, father’s and mother’s age at childbirth, father’s income, education and marital status at childbirth, mother’s pregestational hypertension and diabetes, mother’s preeclampsia, eclampsia, gestational hypertension and diabetes, and mother’s smoking status during pregnancy.

^e^ includes infants of fathers with obesity registered from 2008 to 2019.

^m^ Includes infants of fathers with hypertension, diabetes or dyslipidaemia registered from 2004 to 2019 and obesity from 2008 to 2019.

eTable2. Associations between father’s cardiometabolic conditions and perinatal mortality in their offspring born 2004-2020, Norway, in the restricted populations (mothers with and without risk factors for offspring perinatal mortality).

| Exposure | | Mothers without risk factors for perinatal mortality ^g^ | | | | Mothers with risk factors for perinatal mortality ^g^ | | | |
| --- | --- | --- | --- | --- | --- | --- | --- | --- | --- |
| Hypertension | | Total (N) | Perinatal Deaths, n (%) | IRR* (95%CI) | IRR** (95%CI) | Total (N) | Perinatal Deaths, n (%) | IRR* (95%CI) | IRR** (95%CI) |
|  | No | 471,016 | 2,689 (0.6) | 1.00 (Reference) | 1.00 (Reference) | 113,106 | 909 (0.8) | 1.00 (Reference) | 1.00 (Reference) |
|  | Yes | 8,878 | 77 (0.9) | 1.50 (1.18, 1.90) | 1.37 (1.09, 1.72) | 2,445 | 22 (0.9) | 1.10 (0.73, 1.66) | 1.05 (0.68, 1.63) |
| Diabetes | |  |  |  |  |  |  |  |  |
|  | No | 471,016 | 2,689 (0.6) | 1.00 (Reference) | 1.00 (Reference) | 113,106 | 909 0.8 | 1.00 (Reference) | 1.00 (Reference) |
|  | Yes | 2,165 | 14 (0.7) | 1.16 (0.72, 2.27) | 1.25 (0.69, 1.95) | 604 | 5 (0.8) | 1.02 (0.43, 2.40) | 1.05 (0.43, 2.56) |
| Dyslipidaemia | |  |  |  |  |  |  |  |  |
|  | No | 471,016 | 2,689 (0.6) | 1.00 (Reference) | 1.00 (Reference) | 113,106 | 909 (0.8) | 1.00 (Reference) | 1.00 (Reference) |
|  | Yes | 2,922 | 11 (0.4) | 0.62 (0.33, 1.18) | 0.65 (0.36, 1.16) | 771 | 6 (0.8) | 0.97 (0.44, 2.18) | 0.94 (0.42, 2.10) |
| Obesity ^e^ | |  |  |  |  |  |  |  |  |
|  | No | 373,152 | 2,054 (0.6) | 1.00 (Reference) | 1.00 (Reference) | 79,105 | 589 (0.7) | 1.00 (Reference) | 1.00 (Reference) |
|  | Yes | 1,205 | 13 (1.1) | 2.06 (1.10, 3.86) | 1.91 (1.06, 3.45) | 466 | 5 (1.1) | 1.40 (0.52, 3.78) | 0.98 (0.32, 3.00) |
| Any CMD ^m^ | |  |  |  |  |  |  |  |  |
|  | No | 471,016 | 2,689 (0.6) | 1.00 (Reference) | 1.00 (Reference) | 113,106 | 909 (0.8) | 1.00 (Reference) | 1.00 (Reference) |
|  | Yes | 17,221 | 126 (0.7) | 1.33 (1.09, 1.63) | 1.27 (1.05, 1.53) | 5,017 | 43 (0.9) | 1.06 (0.78, 1.43) | 0.99 (0.72, 1.36) |
|  | Boys | 8,734 | 80 (0.9) | 1.54 (1.22, 1.94) | 1.38 (1.10, 1.73) | 2,583 | 21 (0.8) | 1.06 (0.69, 1.63) | 0.99 (0.63, 1.55) |
|  | Girls | 8,482 | 41 (0.5) | 1.02 (0.74, 1.43) | 1.05 (0.78, 1.40) | 2,430 | 18 (0.7) | 1.04 (0.65, 1.65) | 0.96 (0.59, 1.56) |

CMD: as defined by medication or diagnosis before/during the year of childbirth

^a^ multilevel random-intercept Poisson regression (father's identification number as the random-intercept)

IRR* crude

IRR** adjusted for birthyear of child, father’s and mother’s age at childbirth, father’s income, education and marital status at childbirth, mother’s pregestational hypertension and diabetes, mother’s preeclampsia, eclampsia, gestational hypertension and diabetes, and mother’s smoking status during pregnancy.

^e^ includes infants of fathers with obesity registered from 2008 to 2019.

^m^ Includes infants of fathers with hypertension, diabetes or dyslipidaemia registered from 2004 to 2019 and obesity from 2008 to 2019.

^g^ Risk factors of perinatal mortality: mother’s pregestational and gestational hypertension including preeclampsia, eclampsia, HELLP syndrome and pregestational and gestational diabetes, smoking status during pregnancy

eTable3. Association between father’s cardiometabolic conditions and stillbirth (from 16 weeks to birth). Norway, 2004-2020

| Exposure | | Total (N) | Stillbirth, n (%) | IRR ^a^ | |
| --- | --- | --- | --- | --- | --- |
| Hypertension | |  |  | IRR * (95%CI) | IRR ** (95%CI) |
|  | No | 678,624 | 3,580 (0.5) | 1.00 (Reference) | 1.00 (Reference) |
|  | Yes | 12,840 | 94 (0.7) | 1.37 (1.11, 1.69) | 1.31 (1.04, 1.63) |
| Diabetes | |  |  |  |  |
|  | No | 678,624 | 3,580 (0.5) | 1.00 (Reference) | 1.00 (Reference) |
|  | Yes | 3,153 | 21 (0.7) | 1.28 (0.82, 1.99) | 1.39 (0.88, 2.19) |
| Dyslipidaemia | |  |  |  |  |
|  | No | 678,624 | 3,580 (0.5) | 1.00 (Reference) | 1.00 (Reference) |
|  | Yes | 4,311 | 21 (0.5) | 0.90 (0.59, 1.37) | 0.71 (0.44, 1.16) |
| Obesity ^e^ | |  |  |  |  |
|  | No | 518,429 | 2,572 0.5 | 1.00 (Reference) | 1.00 (Reference) |
|  | Yes | 1,881 | 18 (1.0) | 1.86 (1.12, 3.10) | 1.64 (0.97, 2.79) |
| Any CMD ^m^ | |  |  |  |  |
|  | No | 678,624 | 3,580 (0.5) | 1.00 (Reference) | 1.00 (Reference) |
|  | Yes | 25,325 | 172 (0.7) | 1.27 (1.08, 1.49) | 1.18 (0.99, 1.39) |
|  | Boys | 12,848 | 93 (0.7) | 1.42 (1.15, 1.76) | 1.36 (1.08, 1.71) |
|  | Girls | 12,459 | 61 (0.5) | 1.17 (0.90, 1.53) | 1.09 (0.83, 1.43) |

CMD: cardiometabolic conditions as defined by medication or diagnosis before/during the year of childbirth

^a^ multilevel random-intercept Poisson regression (father's identification number as the random-intercept)

IRR* crude

IRR** adjusted for birthyear of child, father’s and mother’s age at childbirth, father’s income, education and marital status at childbirth, mother’s pregestational hypertension and diabetes, mother’s preeclampsia, eclampsia, gestational hypertension and diabetes, and mother’s smoking status during pregnancy.

^e^ includes infants of fathers with obesity registered from 2008 to 2019.

^m^ Includes infants of fathers with hypertension, diabetes or dyslipidaemia registered from 2004 to 2019 and obesity from 2008 to 2019.

eTable4. Association between father’s cardiometabolic conditions and Neonatal Mortality. Norway, 2004-2020

| Exposure | | Total (N) | Neonatal mortality, n (%) | IRR ^a^ | |
| --- | --- | --- | --- | --- | --- |
| Hypertension | |  |  | IRR* (95%CI) | IRR** (95%CI) |
|  | No | 675,044 | 1,054 (0.2) | 1.00 (Reference) | 1.00 (Reference) |
|  | Yes | 12,746 | 29 (0.2) | 1.20 (0.78, 1.84) | 1.23 (0.80, 1.89) |
| Diabetes | |  |  |  |  |
|  | No | 675,044 | 1,054 (0.2) | 1.00 (Reference) | 1.00 (Reference) |
|  | Yes | 3,132 | 2 (0.1) | 0.41 (0.10, 1.64) | 0.44 (0.11, 1.78) |
| Dyslipidaemia | |  |  |  |  |
|  | No | 675,044 | 1,054 (0.2) | 1.00 (Reference) | 1.00 (Reference) |
|  | Yes | 4,290 | 7 (0.2) | 1.03 (0.49, 2.13) | 0.88 (0.39, 1.99) |
| Obesity ^e^ | |  |  |  |  |
|  | No | 518,429 | 783 (0.2) | 1.00 (Reference) | 1.00 (Reference) |
|  | Yes | 1,881 | 3 (0.2) | 1.08 (0.34, 3.36) | 1.11 (0.35, 3.54) |
| Any CMD ^m^ | |  |  |  |  |
|  | No | 675,044 | 1,054 (0.2) | 1.00 (Reference) | 1.00 (Reference) |
|  | Yes | 25,153 | 38 (0.2) | 0.96 (0.69, 1.35) | 0.95 (0.68, 1.33) |
|  | Boys | 12,755 | 26 (0.2) | 1.13 (0.75, 1.72) | 1.11 (0.73, 1.67) |
|  | Girls | 12,398 | 12 (0.1) | 0.73 (0.41, 1.29) | 0.74 (0.41, 1.33) |

CMD: cardiometabolic conditions as defined by medication or diagnosis before/during the year of childbirth

^a^ multilevel random-intercept Poisson regression (father's identification number as the random-intercept)

IRR* crude

IRR** adjusted for birthyear of child, father’s and mother’s age at childbirth, father’s income, education and marital status at childbirth, mother’s pregestational hypertension and diabetes, mother’s preeclampsia, eclampsia, gestational hypertension and diabetes, and mother’s smoking status during pregnancy.

^e^ includes infants of fathers with obesity registered from 2008 to 2019.

^m^ Includes infants of fathers with hypertension, diabetes or dyslipidaemia registered from 2004 to 2019 and obesity from 2008 to 2019.

eTable5. Association between father’s cardiometabolic conditions and late miscarriage (from 16 to 20 weeks). Norway, 2004-2020

| Exposure | | Total (N) | Spontaneous abortion, n (%) | IRR ^a^ | |
| --- | --- | --- | --- | --- | --- |
| Hypertension | |  |  | IRR* (95%CI) | IRR** (95%CI) |
|  | No | 676,302 | 1,449 (0.2) | 1.00 (Reference) | 1.00 (Reference) |
|  | Yes | 12,776 | 38 (0.3) | 1.36 (0.96, 1.91) | 1.34 (0.93, 1.94) |
| Diabetes | |  |  |  |  |
|  | No | 676,302 | 1,449 (0.2) | 1.00 (Reference) | 1.00 (Reference) |
|  | Yes | 3,139 | 9 (0.3) | 1.37 (0.67, 2.79) | 1.72 (0.83, 3.54) |
| Dyslipidaemia | |  |  |  |  |
|  | No | 676,302 | 1,449 (0.2) | 1.00 (Reference) | 1.00 (Reference) |
|  | Yes | 4,297 | 7 (0.2) | 0.75 (0.36, 1.59) | 0.32 (0.10, 1.00) |
| Obesity ^e^ | |  |  |  |  |
|  | No | 518,256 | 1,019 (0.2) | 1.00 (Reference) | 1.00 (Reference) |
|  | Yes | 1,881 | 8 (0.4) | 1.89 (0.87, 4.12) | 1.81 (0.79, 4.12) |
| Any CMD ^m^ | |  |  |  |  |
|  | No | 676,302 | 1,449 (0.2) | 1.00 (Reference) | 1.00 (Reference) |
|  | Yes | 25,213 | 72 (0.3) | 1.30 (1.01, 1.68) | 1.21 (0.91, 1.61) |
|  | Boys | 12,791 | 41 (0.3) | 1.61 (1.15, 2.25) | 1.56 (1.09, 2.25) |
|  | Girls | 12,405 | 14 (0.1) | 0.99 (0.55, 1.79) | 1.02 (0.56, 1.84) |

CMD: cardiometabolic conditions as defined by medication or diagnosis before/during the year of childbirth

^a^ multilevel random-intercept Poisson regression (father's identification number as the random-intercept)

IRR* crude

IRR** adjusted for birthyear of child, father’s and mother’s age at childbirth, father’s income, education and marital status at childbirth, mother’s pregestational hypertension and diabetes, mother’s preeclampsia, eclampsia, gestational hypertension and diabetes, and mother’s smoking status during pregnancy.

^e^ includes infants of fathers with obesity registered from 2008 to 2019.

^m^ Includes infants of fathers with hypertension, diabetes or dyslipidaemia registered from 2004 to 2019 and obesity from 2008 to 2019.

eTable6. Association between father’s cardiometabolic conditions and Stillbirth (from 22 weeks to birth). Norway, 2004-2020

| Exposure | | Total (N) | Stillbirth,  n (%) | IRR ^a^ | |
| --- | --- | --- | --- | --- | --- |
| Hypertension | |  |  | IRR* (95%CI) | IRR** (95%CI) |
|  | No | 676,983 | 2,130 (0.3) | 1.00 (Reference) | 1.00 (Reference) |
|  | Yes | 12,795 | 57 (0.5) | 1.40 (1.08, 1.83) | 1.31 (1.00, 1.73) |
| Diabetes | |  |  |  |  |
|  | No | 676,983 | 2,130 (0.3) | 1.00 (Reference) | 1.00 (Reference) |
|  | Yes | 3,142 | 12 (0.4) | 1.22 (0.69, 2.13) | 1.19 (0.66, 2.14) |
| Dyslipidaemia | |  |  |  |  |
|  | No | 676,983 | 2,130 (0.3) | 1.00 (Reference) | 1.00 (Reference) |
|  | Yes | 4,304 | 14 (0.3) | 1.01 (0.60, 1.70) | 0.98 (0.58, 1.64) |
| Obesity ^e^ | |  |  |  |  |
|  | No | 518,256 | 1,559 (0.3) | 1.00 (Reference) | 1.00 (Reference) |
|  | Yes | 1,881 | 10 (0.5) | 1.77 (0.90, 3.45) | 1.42 (0.67, 3.03) |
| Any CMD ^m^ | |  |  |  |  |
|  | No | 676,983 | 2,130 (0.3) | 1.00 (Reference) | 1.00 (Reference) |
|  | Yes | 25,242 | 101 (0.4) | 1.26 (1.03, 1.54) | 1.17 (0.94, 1.44) |
|  | Boys | 12,802 | 52 (0.4) | 1.30 (0.99, 1.72) | 1.24 (0.92, 1.66) |
|  | Girls | 12,439 | 48 (0.4) | 1.27 (0.95, 1.69) | 1.13 (0.83, 1.53) |

CMD: cardiometabolic conditions as defined by medication or diagnosis before/during the year of childbirth

^a^ multilevel random-intercept Poisson regression (father's identification number as the random-intercept)

IRR* crude

IRR** adjusted for birthyear of child, father’s and mother’s age at childbirth, father’s income, education and marital status at childbirth, mother’s pregestational hypertension and diabetes, mother’s preeclampsia, eclampsia, gestational hypertension and diabetes, and mother’s smoking status during pregnancy.

^e^ includes infants of fathers with obesity registered from 2008 to 2019.

^m^ Includes infants of fathers with hypertension, diabetes or dyslipidaemia registered from 2004 to 2019 and obesity from 2008 to 2019.

eTable7. Association between father’s cardiometabolic conditions and offspring perinatal mortality. Norway, 2004-2020

| Exposure | | Total (N) ^z^ | Perinatal Deaths, n (%) | IRR ^a, z^ | | Total (N) ^v^ | Perinatal Deaths, n (%) | IRR ^a, v^ | |
| --- | --- | --- | --- | --- | --- | --- | --- | --- | --- |
| Hypertension | |  |  | IRR* (95%CI) | IRR** (95%CI) |  |  | IRR* (95%CI) | IRR** (95%CI) |
|  | No | 680,377 | 4,628 (0.7) | 1.00 (Reference) | 1.00 (Reference) | 678,432 | 4,617 (0.7) | 1.00 (Reference) | 1.00 (Reference) |
|  | Yes | 10,701 | 93 (0.9) | 1.27 (1.02, 1.58) | 1.20 (0.96, 1.50) | 12,833 | 118 | 1.33 (1.10, 1.60) | 1.29 (1.05, 1.57) |
| Diabetes | |  |  |  |  |  |  |  |  |
|  | No | 682,332 | 4,655 (0.7) | 1.00 (Reference) | 1.00 (Reference) | 678,432 | 4,617 (0.7) | 1.00 (Reference) | 1.00 (Reference) |
|  | Yes | 2,768 | 24 (0.9) | 1.28 (0.84, 1.96) | 1.40 (0.92, 2.15) | 3,151 | 23 (0.7) | 1.09 (0.71, 1.65) | 1.16 (0.75, 1.79) |
| Dyslipidaemia | |  |  |  |  |  |  |  |  |
|  | No | 681,723 | 3,373 (0.7) | 1.00 (Reference) | 1.00 (Reference) | 678,432 | 4,617 (0.7) | 1.00 (Reference) | 1.00 (Reference) |
|  | Yes | 3,372 | 22 (0.7) | 0.93 (0.62, 1.40) | 0.80 (0.50, 1.26) | 4,311 | 28 (0.7) | 0.93 (0.64, 1.33) | 0.75 (0.49, 1.14) |
| Obesity ^e^ | |  |  |  |  |  |  |  |  |
|  | No | 521,666 | 3,382 (0.7) | 1.00 (Reference) | 1.00 (Reference) | 518,256 | 3,346 (0.7) | 1.00 (Reference) | 1.00 (Reference) |
|  | Yes | 1,604 | 18 (1.1) | 1.70 (1.03, 2.83) | 1.50 (0.89, 2.55) | 1,881 | 21 (1.1) | 1.69 (1.06, 2.68) | 1.52 (0.94, 2.47) |
| Any CMD ^m^ | |  |  |  |  |  |  |  |  |
|  | No | 682,894 | 4,657 (0.7) | 1.00 (Reference) | 1.00 (Reference) | 678,432 | 4,617 (0.7) | 1.00 (Reference) | 1.00 (Reference) |
|  | Yes | 20,852 | 170 (0.8) | 1.18 (1.00, 1.38) | 1.10 (0.93, 1.30) | 25,314 | 210 (0.8) | 1.20 (1.04, 1.38) | 1.12 (0.96, 1.31) |
|  | Boys | 10,566 | 94 (0.9) | 1.29 (1.04, 1.60) | 1.25 (1.00, 1.56) | 12,843 | 119 (0.9) | 1.35 (1.11, 1.63) | 1.29 (1.06, 1.58) |
|  | Girls | 10,270 | 60 (0.6) | 1.06 (0.81, 1.40) | 0.99 (0.76, 1.31) | 12,453 | 73 (0.6) | 1.06 (0.83, 1.36) | 1.01 (0.78, 1.29) |

CMD: cardiometabolic conditions as defined by medication or diagnosis before/during the year of childbirth

^a^ multilevel random-intercept Poisson regression (father's identification number as the random-intercept)

IRR* crude

IRR** adjusted for birthyear of child, father’s and mother’s age at childbirth, father’s income, education and marital status at childbirth, mother’s pregestational hypertension and diabetes, mother’s preeclampsia, eclampsia, gestational hypertension and diabetes, and mother’s smoking status during pregnancy.

^e^ includes infants of fathers with obesity registered from 2008 to 2019.

^m^ Includes infants of fathers with hypertension, diabetes or dyslipidaemia registered from 2004 to 2019 and obesity from 2008 to 2019.

^Z^ diagnosis of paternal cardiometabolic conditions before the birthyear of child

^v^ diagnosis of paternal cardiometabolic conditions before and during the birthyear of child

eTable8. Characteristics of perinatal losses in Norway, 2004-2020, by father’s cardiometabolic conditions.

|  | | Cardiometabolic conditions, total population | | |
| --- | --- | --- | --- | --- |
|  | | Total (N) | No, n (%) | Yes, n (%) |
| Number of dead Infants, n (%) | | 4,827 (0.7) | 4,617 (0.7) | 210 (0.8) |
| Birthweight (g), mean (SD) | | 1,417 (1,405) | 1,415 (1,328) | 1,460 (1,405) |
| Gestational Age, n (%) | |  |  |  |
|  | Extremely Preterm (≤31) | 2,556 (53.2) | 2,453 (53.1) | 113 (53.8) |
|  | Preterm (32-36) | 588 (12.2) | 565 (12.2) | 23 (11.0) |
|  | Term (37-41) | 1,101 (22.8) | 1,052 (22.8) | 49 (23.3) |
|  | Post term (≥42) | 38 (0.8) | 36 (0.8) | 2 (1.0) |
|  | Missing | 534 (11.1) | 511 (11.1) | 23 (11.0) |
| Weight for gestation, n (%) | |  |  |  |
|  | SGA 2.5 | 767 (15.9) | 736 (15.9) | 31 (14.8) |
|  | SGA 10 | 1,339 (31.4) | 1,273 (27.6) | 66 (31.4) |
|  | LGA 90 | 230 (4.8) | 220 (4.8) | 10 (4.8) |

eTable9. Association between father’s cardiometabolic conditions and offspring perinatal mortality. Norway, 2004-2020

| Exposure | | Total (N) ^q^ | Perinatal Deaths, n (%) | IRR ^a, q^ | | Total (N) ^p^ | Perinatal Deaths, n (%) | IRR ^a, p^ | |
| --- | --- | --- | --- | --- | --- | --- | --- | --- | --- |
| Hypertension | |  |  | IRR* (95%CI) | IRR** (95%CI) |  |  | IRR* (95%CI) | IRR** (95%CI) |
|  | No | 676,028 | 4,603 (0.7) | 1.00 (Reference) | 1.00 (Reference) | 678,432 | 4,617 (0.7) | 1.00 (Reference) | 1.00 (Reference) |
|  | Yes | 14,018 | 124 (0.9) | 1.22 (0.99, 1.51) | 1.27 (1.05, 1.53) | 12,833 | 118 | 1.33 (1.10, 1.60) | 1.29 (1.06, 1.58) |
| Diabetes | |  |  |  |  |  |  |  |  |
|  | No | 676,028 | 4,603 (0.7) | 1.00 (Reference) | 1.00 (Reference) | 678,432 | 4,617 (0.7) | 1.00 (Reference) | 1.00 (Reference) |
|  | Yes | 4,116 | 30 (0.7) | 1.12 (0.76, 1.64) | 1.13 (0.78, 1.64) | 3,151 | 23 (0.7) | 1.09 (0.71, 1.65) | 1.16 (0.75; 1.79) |
| Dyslipidaemia | |  |  |  |  |  |  |  |  |
|  | No | 676,028 | 4,603 (0.7) | 1.00 (Reference) | 1.00 (Reference) | 678,432 | 4,617 (0.7) | 1.00 (Reference) | 1.00 (Reference) |
|  | Yes | 4,137 | 25 (0.6) | 0.88 (0.60, 1.29) | 0.88 (0.58, 1.33) | 4,311 | 28 (0.7) | 0.93 (0.64, 1.33) | 0.76 (0.50; 1.15) |
| Obesity ^e^ | |  |  |  |  |  |  |  |  |
|  | No | 515,852 | 3,332 (0.7) | 1.00 (Reference) | 1.00 (Reference) | 518,256 | 3,346 (0.7) | 1.00 (Reference) | 1.00 (Reference) |
|  | Yes | 1,754 | 17 (1.0) | 1.42 (0.81, 2.50) | 1.35 (0.80, 2.28) | 1,881 | 21 (1.1) | 1.69 (1.06, 2.68) | 1.53 (0.94; 2.48) |
| Any CMD ^m^ | |  |  |  |  |  |  |  |  |
|  | No | 676,028 | 4,603 (0.7) | 1.00 (Reference) | 1.00 (Reference) | 678,432 | 4,617 (0.7) | 1.00 (Reference) | 1.00 (Reference) |
|  | Yes | 27,718 | 224 (0.8) | 1.13 (0.97, 1.32) | 1.14 (0.99, 1.31) | 25,314 | 210 (0.8) | 1.20 (1.04, 1.38) | 1.13 (0.97; 1.32) |
|  | Boys | 14,114 | 124 (0.9) | 1.23 (1.00, 1.51) | 1.25 (1.03, 1.52) | 12,843 | 119 (0.9) | 1.35 (1.11, 1.63) | 1.31 (1.07; 1.61) |
|  | Girls | 13,582 | 78 (0.6) | 1.02 (0.79, 1.33) | 1.01 (0.79, 1.27) | 12,453 | 73 (0.6) | 1.06 (0.83, 1.36) | 1.00 (0.78; 1.29) |

CMD: cardiometabolic conditions as defined by medication or diagnosis before/during the year of childbirth

^a^ multilevel random-intercept Poisson regression (father's identification number as the random-intercept)

IRR* crude

IRR** adjusted for birthyear of child, father’s and mother’s age at childbirth, father’s income, education and marital status at childbirth, mother’s pregestational hypertension and diabetes, mother’s preeclampsia, eclampsia, gestational hypertension and diabetes, and mother’s smoking status during pregnancy.

^e^ includes infants of fathers with obesity registered from 2008 to 2019.

^m^ Includes infants of fathers with hypertension, diabetes or dyslipidaemia registered from 2004 to 2019 and obesity from 2008 to 2019.

^q^ Diagnosis based on combination of Norwegian Patient Registry and Norwegian Prescription Database

^P^ Diagnosis based on Norwegian Prescription Database only
